# Tripterygium Glycosides Combined with Leflunomide for Rheumatoid Arthritis: A Systematic Review and Meta-Analysis

**DOI:** 10.1155/2020/1230320

**Published:** 2020-05-08

**Authors:** Yi-Jing Yang, Ying Deng, Lin-Li Liao, Jun Peng, Qing-Hua Peng, Yu-Hui Qin

**Affiliations:** ^1^Hunan University of Chinese Medicine, Changsha 410208, China; ^2^The First Hospital of Hunan University of Chinese Medicine, Changsha, Hunan 410007, China

## Abstract

**Objective:**

To undertake an overview on the overall effects of Tripterygium glycosides (TG) combined with Leflunomide (LEF) for rheumatoid arthritis (RA).

**Methods:**

We searched electronic databases from database establishment time to December 1, 2019. The clinical trial data of TG combined with LEF (trial group) and control group in the treatment of RA were collected. The Cochrane system was used to evaluate the quality of the literature. RevMan 5.3 software was used to conduct a meta-analysis of the eligible studies.

**Results:**

A total of 12 randomized controlled trials (RCTs) involving 834 patients with RA were included in this study. The meta-analysis results showed that morning stiffness (mean difference (MD) = −0.29, 95% confidential interval (CI) (−0.45, −0.12), *P*=0.0005), tender joint count (MD = −1.51, 95% CI (−2.20, −0.83), *P*=0.0001), swollen joint count (MD = -1.24, 95% CI (−1.59, −0.88), *P*=0.0001), erythrocyte sedimentation rate (MD = −7.26, 95% CI (−9.92, −4.61), *P*=0.0001), C-reactive protein (MD = −4.04, 95% CI (−4.93, −3.14), *P*=0.0001), and rheumatoid factor (MD = −50.88, 95% CI (−72.30, −29.45), *P* = 0.0001) in the trial groups were lower than those in the control groups. The total effective rate in the trial group was better than that in the control group (risk ratio (RR) = 1.20, 95% CI (1.13, 1.28), *P*=0.00001). However, there was no significant difference of adverse events (RR = 0.83, 95% CI (0.61, 1.13), *P*=0.23) while comparing the trial groups with the control groups.

**Conclusion:**

Our results were found to be superior but limited evidence on the effectiveness of TG combined with LEF in the treatment of RA is available.

## 1. Introduction

Rheumatoid arthritis (RA) is the most common inflammatory arthritis of unknown etiology that primarily affects joints, with a prevalence of up to 1% worldwide [1, 2]. The most common factors affecting RA are environment, age, female, and those with a family history [3, 4]. Currently, there are several drugs used in the treatment of RA, including nonsteroidal anti-inflammatory drugs (NSAIDs), disease-modifying antirheumatic drugs (DMARDs), corticosteroids, and biological agents [5]. However, the specific therapeutic treatment for RA is still a dilemma in the modern medicine.

Tripterygium glycoside (TG) is a Chinese patent medicine and active compound extracted from the roots of a Chinese herb named *Tripterygium wilfordii*. TG is a novel anti-inflammatory agent and immunosuppressant, especially for nephrotic syndrome, diabetic nephropathy, and RA. Leflunomide (LEF, also named Arava) is an immunosuppressive drug that works by inhibiting dihydroorotate dehydrogenase and is used in active moderate-to-severe rheumatoid arthritis [6]. Recently, several single-centers, prospective, randomized controlled trials indicate that addition of TG might achieve better effectiveness than monotherapy of LEF in RA. In this study, we performed a systematic analysis of the current available evidence to estimate the efficacy of TG combined with LEF for RA. This review was conducted according to the PRISMA statement [7].

## 2. Methods

### 2.1. Literature Search

We carried on a systematic search in databases including PubMed, Cochrane Library, Medline, Chinese Scientific Journal Database (VIP) Journal, China National Knowledge Infrastructure (CNKI), and Wan fang database without language limits up to December 1, 2019. The following domains of terms were used in combination, Tripterygium glycosides or glycosides of *Tripterygium wilfordii* or Tripterygium and Leflunomide or Arava and rheumatoid arthritis or RA.

### 2.2. Inclusion and Exclusion Criteria

The included trials were evaluated by the following criteria: (1) study design: randomized controlled trials (RCTs); (2) patients receiving a diagnosis of RA according to 2010 rheumatoid arthritis classification criteria: an American College of Rheumatology/European League against Rheumatism collaborative initiative [2]; there was no limit on age, gender, race, duration of suffering, and severity of the disease; (3) therapy of combined TG with LEF was chosen as the trial group for treatment of RA, and the control group received LEF or other anti-RA drugs. Both the trial and control groups were given the same basic therapy.

The exclusion criteria were as follows: (1) non-RCTs, (2) patients did not meet the diagnostic criteria of RA, (3) low-quality clinical trials, (4) duplicated or incomplete publications, and (5) letters, comments, reviews, and animal researches.

### 2.3. Data Extraction Strategy

Abstracts and article titles of each included study were independently reviewed by pairs of authors (Yi-Jing Yang and Ying Deng). All disagreements were settled through discussion with all of the authors. Clinical characteristics of included publications were summarized, and the main outcomes and adverse effects (AEs) of each work were also pulled out.

### 2.4. Statistical Data Analysis

Meta-analysis was performed by using RevMan (version 5.3) software. If there were multiple measurement data, only the most long-term treatment data were adopted in the analysis. The mean differences (MD) and risk ratios (RR) were used for continuous variables and dichotomous variables, respectively. Pooling estimates and their 95% confidential intervals (95% CI) were calculated. *P* < 0.05 was considered statistically significant. A chi-squared test with *P* value and the *I*^2^ statistic were used to quantify the statistical heterogeneity between studies. If no heterogeneity between studies was observed (*P* > 0.1 or *I*^2^ < 50%), the fixed effect model was used for the analysis; otherwise, the random effect model was used. Forest plots displayed summary weighted estimates and the funnel plots could be applied to assess the publication biases. The publication biases were evaluated by the funnel plot.

## 3. Results

### 3.1. Literature Search

**Figure 1:**
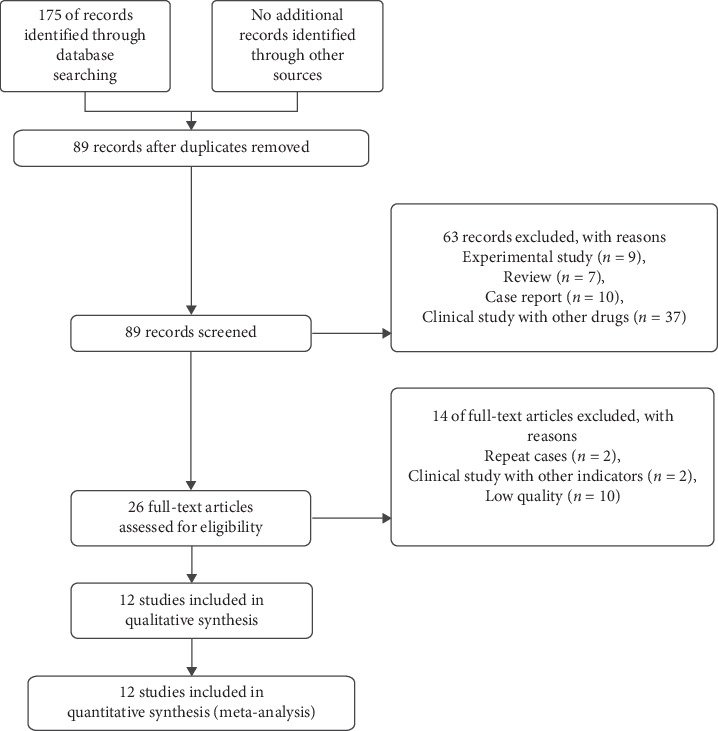
Flow diagram for literature searching.

Initially, 175 publications were identified through searching databases. After removing duplicates, the titles and abstracts of 89 potentially relevant articles were reviewed. We browsed the titles and abstracts, and 26 articles were reviewed with full texts. Finally, 12 completed RCTs (*n* = 834) were included in the meta-analysis. The literature search process is illustrated as a flow diagram in Figure 1. All of the included subjects were issued between 2009 and 2019. The distribution of age and gender had no significant differences in all subjects, and the treatment duration ranged from 1 month to 6 months [8–19]. The main characteristics of the included studies are summarized in Table 1.

### 3.2. Study Quality Assessment

**Figure 2:**
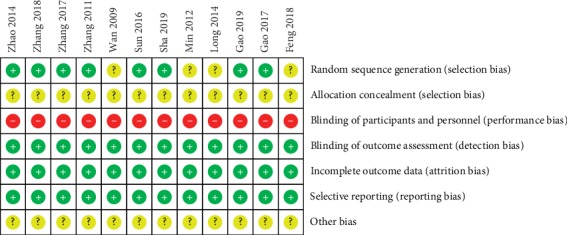
Risk of bias summary.

The methodological quality of the included studies was generally poor (Table 2, Figure 2). Altogether the 12 studies were RCTs, and eight of these studies are mentioned in the appropriate method of random sequence generation. The allocation concealment was not reported in all articles. Moreover, none of the included studies mentioned blinding of participants and personnel as well as blinding of outcome assessment. The withdrawals and dropouts were not noted in all the works.

### 3.3. Results of Meta-Analysis

#### 3.3.1. Clinical Treatment Efficacy

**Figure 3:**
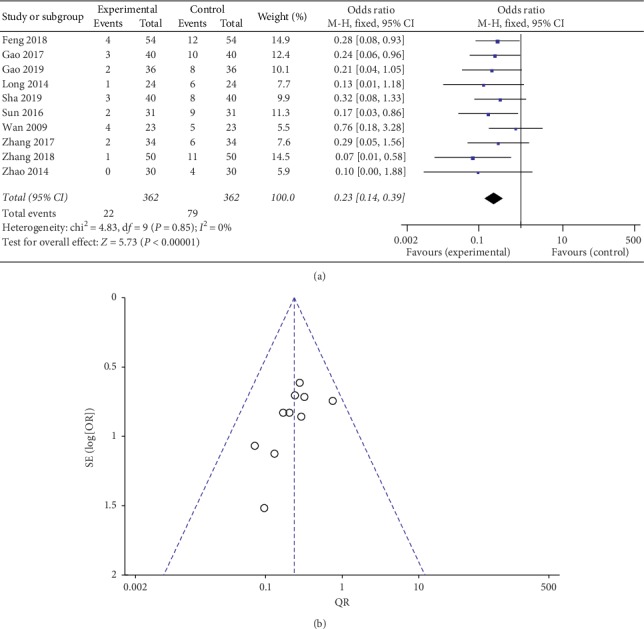
Forest plot (a) and funnel plot (b) of Tripterygium glycosides combined with Leflunomide in the treatment of rheumatoid arthritis for total efficacy.

Nine studies reported the clinical intervention of TG combined with LEF in the treatment of RA, seven of those studies were compared with LEF monotherapy, while two studies, respectively, were compared with methotrexate or methotrexate plus sulfasalazine. The other three subjects described the clinical intervention of TG combined with LEF plus methotrexate compared with Leflunomide plus methotrexate in the treatment of RA. Zhang, 2011, and Min, 2012, did not describe the clinical efficacy of therapy. The other ten studies reported the total clinical efficacy of combination therapy compared with control therapy. Significant statistical heterogeneity was not found in the meta-analysis (chi^2^ = 2.80, *I*^2^ = 0%, *P*=0.97 > 0.05), thus the risk ratio (RR) was pooled by a fixed effect model; results indicated a better clinical efficacy of combination of TG and LEF therapy than the control therapy (RR = 1.20, 95% confidence interval (CI) (1.13, 1.28), *P* < 0.00001) (Figure 3). The funnel plot was carried out, and there was no outlier in the result. The independent research effect points on the funnel plot are not distributed symmetrically, and a certain heterogeneity was inevitable due to the small samples and different drugs.

### 3.4. Morning Stiffness

**Figure 4:**
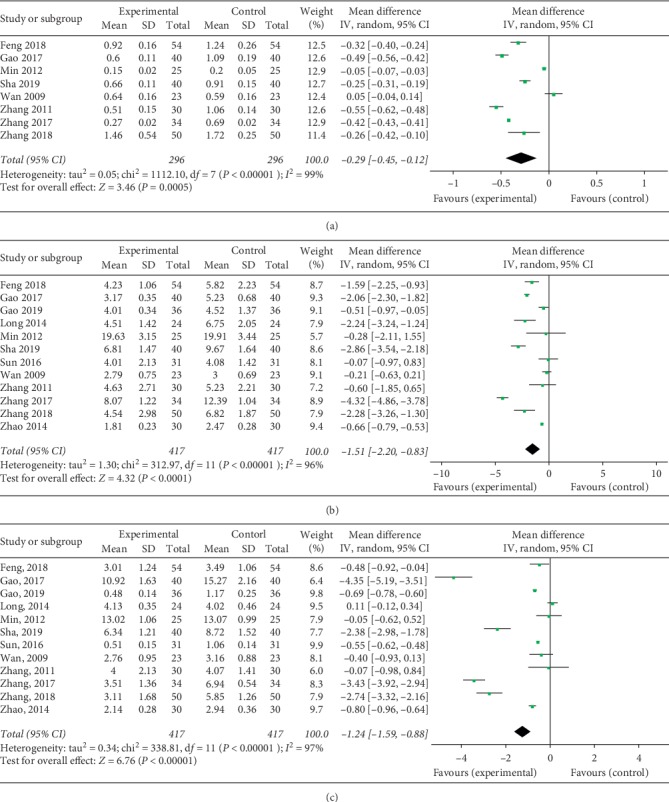
Forest plot of Tripterygium glycosides combined with Leflunomide in the treatment of rheumatoid arthritis for morning stiffness (a), tender joint count (b), and swollen joint count (c).

Eight of the 12 included studies investigated the duration of morning stiffness of posttreatment. As significant heterogeneity existed across the studies (chi^2^ = 1112.10, *I*^2^ = 99%, *P* < 0.00001), the random effect model was used for the analysis. The meta-analysis demonstrated that the therapeutic method of TG combined with LEF significantly decreased the duration of morning stiffness in RA treatment when compared with control treatment (Mean difference (MD) = −0.29, 95% CI (−0.45, −0.12), *P* < 0.0005) (Figure 4(a)).

### 3.5. Tender Joint Count

All the 12 included studies inquired into tender joint count after the treatment. Because significant statistical heterogeneity was found in the meta-analysis (chi^2^ = 312.97, *I*^2^ = 96%, *P* < 0.00001), the random effect model was used for the analysis. The result suggested that a therapeutic method of TG combined with LEF significantly alleviated tender joint count in RA treatment when compared with control treatment (MD = −1.51, 95% CI (−2.20, −0.83), *P* < 0.00001) (Figure 4(b)).

### 3.6. Swollen Joint Count

All the 12 included studies surveyed the swollen joint count of posttreatment. Owing to significant statistical heterogeneity presented in the studies (chi^2^ = 338.81, *I*^2^ = 97%, *P* < 0.00001), the random effect model was employed for the analysis. Results implicated that the therapeutic method of TG combined with LEF significantly relieved swollen joint count in RA treatment when compared with control treatment (MD = −1.24, 95% CI (−1.59, −0.88), *P* < 0.0001) (Figure 4(c)).

### 3.7. Erythrocyte Sedimentation Rate

**Figure 5:**
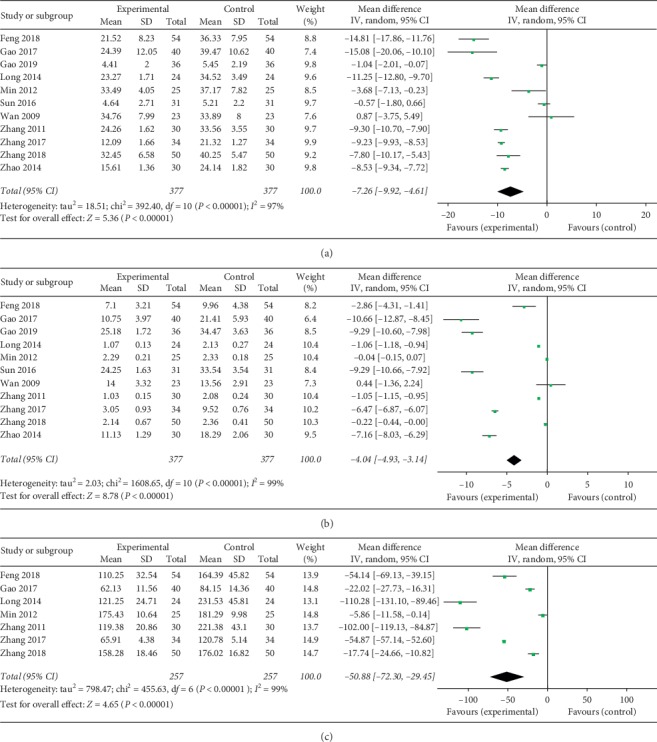
Forest plot of Tripterygium glycosides combined with Leflunomide in the treatment of rheumatoid arthritis for erythrocyte sedimentation rate (a), C-reactive protein (b), and rheumatoid factor (c).

Eleven included studies researched the erythrocyte sedimentation rate (ESR) of patients with RA after the completion of treatment. There was a significant heterogeneity in this analysis (chi^2^ = 392.40, *I*^2^ = 97%, *P* < 0.00001), and the random effect model was employed for the analysis. The results suggested that a therapeutic method of TG combined with LEF significantly decreased ESR in RA treatment when compared with control treatment (MD = −7.26, 95% CI (−9.92, −4.61), *P* < 0.0001) (Figure 5(a)).

### 3.8. C-Reactive Protein

Eleven studies compared C-reactive protein (CRP) between the trial groups and control groups. A significant heterogeneity existed across the studies (chi^2^ = 1608.65, *I*^2^ = 99%, *P* < 0.00001), and the overall effect was calculated from a random effect model. The result suggested that a therapeutic method of TG combined with LEF significantly decreased CRP in RA treatment when compared with control treatment (MD = −4.04, 95% CI (−4.93, −3.14), *P* < 0.0001) (Figure 5(b)).

### 3.9. Rheumatoid Factor Test

Seven trials inspected rheumatoid factor (RF) between the test groups and control groups. There was significant heterogeneity existed across the studies (chi^2^ = 455.63, *I*^2^ = 99%, *P* < 0.00001), and the overall effect was pooled by a random effect model. The result suggested that a therapeutic method of TG combined with LEF significantly decreased RF in RA treatment when compared with control treatment (MD = −50.88, 95% CI (−72.30, −29.45), *P* < 0.0001) (Figure 5(c)).

### 3.10. Interleukin 1

**Figure 6:**
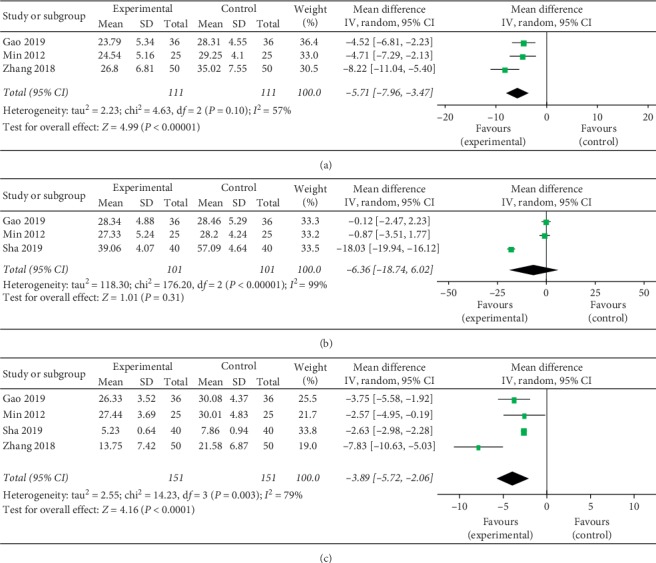
Forest plot of Tripterygium glycosides combined with Leflunomide in the treatment of rheumatoid arthritis for IL-1 (a), IL-6 (b), and TNF-*α* (c).

Three trials compared IL-1 between the trial groups and control groups. There was significant heterogeneity in this analysis (*I*^2^ = 57%, *P*=0.1), and a random effect model was applied. The results suggested that a therapeutic method of TG combined with LEF significantly declines the level of IL-1 in RA treatment when compared with control treatment (MD = −5.71, 95% CI (−7.96, −3.47), *P* < 0.0001) (Figure 6(a)).

### 3.11. Interleukin 6

Three trials compared IL-6 between the trial groups and control groups. There was significant heterogeneity in this analysis (*I*^2^ = 57%, *P*=0.1), and a random effect model was taken up. The results suggested that IL-6 was not different between the trial groups and control groups in RA treatment (MD = −6.36, 95% CI (−18.74, 6.02), *P* < 0.0001) (Figure 6(b)).

### 3.12. Tumor Necrosis Factor-Alpha

Four trials compared TNF-*α* between the trial groups and control groups. There was significant heterogeneity in this analysis (*I*^2^ = 79%, *P*=0.003), and a random effect model was applied. The results suggested that a therapeutic method of TG combined with LEF significantly declines the level of TNF-*α* in RA treatment when compared with control treatment (MD = −5.71, 95% CI (−7.96, −3.47), *P* < 0.0001) (Figure 6(c)).

### 3.13. Adverse Events

**Figure 7:**
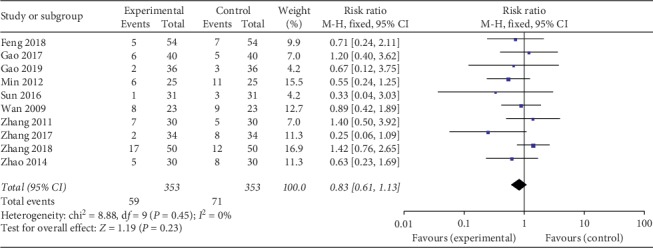
Forest plot of Tripterygium glycosides combined with Leflunomide in the treatment of rheumatoid arthritis for adverse events.

We calculated the overall risk ratio for AEs associated with the therapeutic method of combining with TG in RA treatment compared with control treatment, and ten trials were included in this analysis. There were 59 (16.7%) AEs in 353 patients in the trial groups, while 71 (20.1%) AEs in 353 patients in the control group. No significant heterogeneity was found in this analysis. There was no significant difference of AEs while comparing the trial groups with the control groups (RR = 0.83, 95% CI (0.61, 1.13), *P*=0.23) (Figure 7).

## 4. Discussion

RA is primarily caused by chronic synovial hyperplasia, which affects bone, and renders patients with different degrees of disability. The principle medicals for RA include nonsteroidal anti-inflammatory drugs, slow-playing antirheumatic drugs, glucocorticoids, and biological agents. Leflunomide, belongs to DMARD for RA and inhibits de novo pyrimidine biosynthesis as effective as methotrexate therapy for patients with active RA and less liver damage [20, 21].

TG is a fat-soluble mixture extracted from the root of the plant *Tripterygium wilfordii* [22]. *Tripterygium wilfordii* has been used in treating RA for long years in traditional Chinese medicine [23]. Studies found that the compound of TG can downregulate the LPS-stimulated NF-*κ*B and MAPK kinase activity in macrophages, inhibit the expression of iNOS, promote its degradation, and reduce NO release [24, 25]. Evidence found that TG inhibited IL-1 and activated spinal cord cells to secrete matrix metalloproteinases MMP-3 and MMP-13, which improves capillary fine cell viability and alleviates joint bone tissue degradation [26]. Research in synovial fibroblasts of arthritis patients suggested that TG has an effect on decreasing NF-*κ*B activity, inhibiting COX-2 and iNOS gene expression, reducing PGE_2_ and NO production, and promoting caspase-3 expression [27, 28].

In the current systematic review, TG is considered to behave like DMARDs and may contribute to the reduction of inflammation, prevent or limit joint damage, and improve physical function in RA patients. Serious adverse events associated with the use of TG have been well documented, and it is noteworthy that hepatotoxicity was rarely reported. However, there is a high incidence of *tuberculosis* and hepatitis B in population of mainland of China. It is particularly important to find antirheumatic drug treatment programs that are suitable for this group of people. In addition, TG has been widely applied by rheumatologists as a standard therapy for RA in Chinese hospitals and has been accepted by patients due to a low cost of treatment.

The primary outcome of this study is clinical efficacy. The study designed overviews of data from 12 RCTs revealed that, compared with the control, the therapeutic method of TG combined with LEF for RA got a better clinical efficacy. The secondary outcomes are clinical symptoms and in the level of serum inflammatory factors. The combination therapy significantly decreased the duration of morning stiffness, alleviated tender joint count, relieved swollen joint count, decreased ESR, CRP, and RF, and declined the levels of IL-1 and TNF-*α*. However, the trial groups were not superior to the control groups in terms of decreasing IL-6 and AEs. The results indicated that combination of TG can reduce the level of serum inflammatory factors, delay the progression of bone damage, and alleviate RA to some extent. On inspection of the funnel plot for the efficacy, the independent research effect points on the funnel plot are not distributed symmetrically and certain heterogeneity was inevitable due to the small samples and different drugs. Nevertheless, considering the differences in clinical heterogeneity and varying levels of statistical heterogeneity, the results were found to be superior but limited evidence on the effectiveness and safety of TG combined with LEF in the treatment of RA was available.

There are several limitations to be taken into account. Foremost, all included studies have a small number of participants or are single-center study. Second, longer-term studies are required to establish the effect of combination treatment. Third, trials were generally of poor quality, and the funnel plot was carried out in clinical treatment efficacy; heterogeneity was inevitable due to the different drugs in both trial groups and control groups. Last, considering that all included trials were performed in China, a possibility exists that negative results were less likely to be published, which might contribute to a bias.

In summary, patients receiving TG combined with LEF therapy may derive an important benefit that was demonstrated by the limited improvement in the clinical signs and symptoms of RA.

## Figures and Tables

**Table 1:** Characteristics of the eligible studies.

Study author, year (study period)	Patients total (Ctrl)	Age/years (Trl) (Ctrl)	Genders M/F total (Trl)	Intervention/drug dosage (Trl) (Ctrl)	Treating duration (M)	Indexes	AEs (Trl) (Ctrl)
Wan, 2009 [8] (2005.11–2008.3)	46 (23)	32 ± 15 (34 ± 12)	6/40 (4/19)	TG + LEF/10 mg, qd + 20 mg, qd (MTX + SASP/10 mg qw + 1 g bid)	3	Efficacy, MS, SJC, TJC, ESR, CRP	8 (9)

Zhang, 2011 [9] (2007.1–2008.12)	60 (30)	66 (66)	N. M.	TG + LEF/20 mg, tid + 20 mg, qd (LEF/20 mg, qd)	6	MS, SJC, TJC, ESR, CRP, RF	7 (5)

Min, 2012 [10] (2009.1–2011.6)	50 (25)	Mean 61.5	12/38	TG + LEF/20 mg, tid + 10 mg, qd (MTX/15 mg, qw)	3	MS, SJC, TJC, ESR, CRP, RF, IL-1, IL-6, TNF-*α*	6 (11)

Long, 2014 [11] (2011.6–2012.6)	48 (24)	67.00 ± 2.47 (68.00 ± 2.95)	21/27	TG + LEF/20 mg, tid + 10 mg, qd (LEF/10 mg, qd)	6	Efficacy, SJC, TJC, ESR, CRP, RF	NM

Zhao, 2014 [12] (2010.7–2013.7)	60 (30)	45.07 ± 20.32 (45.10 ± 20.21)	10/50	TG + LEF + MTX/N. M. + 10 mg, qd + 7.5 mg, qw (LEF + MTX/10 mg, qd + 7.5 mg, qw)	3	Efficacy, MS, SJC, TJC, ESR, CRP	5 (8)

Sun, 2016 [13] (2014.7–2015.7)	62 (31)	62.11 ± 3.02 (56.56 ± 2.33)	32/30 (17/14)	TG + LEF/20 mg, tid + 5 mg, bid (LEF/5 mg, bid)	6	Efficacy, SJC, TJC, ESR, CRP	1 (3)

Gao, 2017 [14] (2015.1–2016.7)	80 (40)	42.35 ± 8.14 (41.67 ± 8.65)	24/56 (11/29)	TG + LEF + MTX/20 mg, tid + 10 mg, qd + 10 mg, qw (MTX/10 mg, qw)	3	Efficacy, MS, SJC, TJC, ESR, CRP, RF	6 (5)

Zhang, 2017 [15] (2014.1–2017.1)	64 (34)	49.90 ± 15.22 (43.56 ± 15.07)	21/47 (10/24)	TG + LEF + MTX/10 mg, tid+10 mg, qd + 10 mg, qw (LEF + MTX/10 mg, qd + 10 mg, qw)	3	Efficacy, MS, SJC, TJC, ESR, CRP, RF	2 (8)

Feng, 2018 [16] (2015.5–2017.5)	108 (54)	70.12 ± 5.13 (72.05 ± 6.24)	55/53 (26/28)	TG + LEF/20 mg, tid + 10 mg, qd (LEF/10 mg, qd)	1	Efficacy, MS, SJC, TJC, ESR, CRP, RF	5 (7)

Zhang, 2018 [17] (2014.3–2016.6)	100 (50)	64.8 ± 11.52	37/63	TG + LEF + MTX/20 mg, tid+20 mg, qd + 10 mg, qw (LEF + MTX/20 mg, qd + 10 mg, qw)	3	Efficacy, MS, SJC, TJC, ESR, CRP, RF, IL-1, 4, 10, TNF-*α*	2 (3)

Gao, 2019 [18] (2012.1–2017.12)	72 (36)	67.9 ± 2.3 (68.7 ± 2.5)	20/16 (42/30)	TG + LEF/20 mg, tid + 5 mg, bid (LEF/5 mg, bid)	6	Efficacy, SJC, TJC, ESR, CRP, IL-1, IL-6, TNF-*α*	2 (3)

Sha, 2019 [19] (2016.10–2018.10)	80 (40)	58.89 ± 6.92 (58.99 ± 6.99)	37/43 (18/22)	TG + LEF/20 mg, tid + 20 mg, qd (LEF/20 mg, qd)	3	Efficacy, MS, SJC, TJC, IL-6, TNF-*α*	NM

AEs: adverse events, Trl: trial group, Ctrl: control group, TG: Tripterygium glycosides, LEF: Leflunomide, MTX: methotrexate, SASP: sulfasalazine, 3M: 3 months, 6M: 6 months, 1M: 1 month, efficacy: clinical treatment efficacy, MS: morning stiffness, SJC: swollen joint count, TJC: tender joint count, ESR: erythrocyte sedimentation rate, CRPC-reactive protein, RF: rheumatoid factor, IL-1: interleukin 1, IL-4: interleukin 4, IL-6: interleukin 6, IL-10: interleukin 10, TNF-*α*: tumor necrosis factor-alpha, NM: not mentioned.

**Table 2:** Quality assessment of the eligible studies.

Studies (author, year)	Randomization	Allocation concealment	Blinding	Incomplete outcome data	Withdrawals and dropouts
Wan Shao-Fen 2009 [8]	Yes	NM	NM	No	NM
Zhang Rong and Le 2011 [9]	Yes, SS	NM	NM	No	NM
Min Jing and Gu 2012 [10]	Yes	NM	NM	No	NM
Long Hong 2014 [11]	Yes	NM	NM	No	NM
Zhao and Zhang 2014 [12]	Yes, SS	NM	NM	No	NM
Sun Fengyan 2016 [13]	Yes, SS	NM	NM	No	NM
Deng-Wen 2017 [14]	Yes, RNT	NM	NM	No	NM
Zhang 2017 [15]	Yes, RNT	NM	NM	No	NM
Feng Yan-Guang and Kun 2018 [16]	Yes	NM	NM	No	NM
Zhang 2018 [17]	Yes, RNT	NM	NM	No	NM
Yang 2019 [18]	Yes, RNT	NM	NM	No	NM
Sha and Yu 2019 [19]	Yes, RNT	NM	NM	No	NM

SS: stratified sampling, RNT: random number table, NM: not mentioned.

## Data Availability

The data supporting this meta-analysis are from the previously reported studies and data sets which have been cited. The processed data are available from Dr. Yi-jing Yang and Dr. Ying Deng upon request.
